# Analysis of EEG entropy during visual evocation of emotion in schizophrenia

**DOI:** 10.1186/s12991-017-0157-z

**Published:** 2017-09-25

**Authors:** Wen-Lin Chu, Min-Wei Huang, Bo-Lin Jian, Kuo-Sheng Cheng

**Affiliations:** 1 Department of Biomedical Engineering, National Cheng Kung University, Tainan, 701 Taiwan; 2 Department of Psychiatry, Chiayi Branch, Taichung Veterans General Hospital, Chia-Yi, 600 Taiwan; 3 Department of Aeronautics and Astronautics, National Cheng Kung University, Tainan, 701 Taiwan

**Keywords:** Electroencephalography, Emotion, Entropy, Schizophrenia, Support vector machine

## Abstract

**Background:**

In this study, the international affective picture system was used to evoke emotion, and then the corresponding signals were collected. The features from different points of brainwaves, frequency, and entropy were used to identify normal, moderately, and markedly ill schizophrenic patients.

**Methods:**

The signals were collected and preprocessed. Then, the signals were separated according to three types of emotions and five frequency bands. Finally, the features were calculated using three different methods of entropy. For classification, the features were divided into different sections and classification using support vector machine (principal components analysis on 95%). Finally, simple regression and correlation analysis between the total scores of positive and negative syndrome scale and features were used.

**Results:**

At first, we observed that to classify normal and markedly ill schizophrenic patients, the identification result was as high as 81.5%, and therefore, we further explored moderately and markedly ill schizophrenic patients. Second, the identification rate in both moderately and markedly ill schizophrenic patient was as high as 79.5%, which at the Fz point signal in high valence low arousal fragments was calculated using the ApEn methods. Finally, the total scores of positive and negative syndrome scale were used to analyze the correlation with the features that were the five frequency bands at the Fz point signal. The results show that the *p* value was less than .001 at the beta wave in the 15–18 Hz frequency range.

## Background

Schizophrenia typically occurs during young adulthood, leading to social deficits in areas such as interpersonal relationships, employment situations, and self-care in patients. The condition requires long-term medication for treatment, which places a significant expenditure burden on families and the health care system [1, 2]. Up to now, many studies have been conducted in the prognosis of schizophrenia [3], which understandably involves a wide range of aspects. However, the results of these studies vary greatly. Although the reliability and comparability of these studies have been increased due to the use of standardized diagnostic interview and symptom evaluation tools, many problems still remain, including the selection of suitable methodologies, patient definition, consistency in description and evaluation tools, sampling, and research design.

The analysis of EEG signals is a noninvasive and nonradioactive tool that can be used for long-term measurements, and therefore plays a very important role in clinical diagnosis. The analysis of EEG signals is used during functional neurological examinations to assist in the diagnosis of brain dysfunctions caused by nonstructural lesions of the brain such as epilepsy [4], dementia [5], and intellectual developmental disorders [6, 7], as well as in research into schizophrenia and other mental disorders [8]. To further understand the response of schizophrenic patients to various types of stimuli, many studies use visual [9, 10] or auditory [8, 11, 12] stimuli to evoke various emotions in schizophrenic patients, and then capture and analyze their EEG signals to determine whether the signals are associated with physiological mechanisms or representative of schizophrenia symptoms. In this study, visual stimulation was used to evoke emotions in normal, moderately, and markedly ill schizophrenic patients, then the corresponding changes in EEG signals were explored to extend the existing body of knowledge in this field of research.

Emotions result from the complex interaction of psychological and physiological phenomena, and the cortex reacts differently according to the type of emotions [13]. Therefore, studies on the elicitation of emotions often use a variety of different methods to induce emotional responses. Gross and Livenson used stimulating fragments from films for emotion induction [14]. Lang et al. recorded the susceptibility of subjects to picture stimulation and objectively developed the scoring criteria for visual complexity and emotional reactions [15]. Palomb et al. induced emotional reactions in subjects using films containing threatening surgery procedures [16]. Kim et al. used colored light, music, and other environmental factors to create different emotional environments [17]. Although subsequent research involved new methods, there is a lack of objective standards of stimulation strength and diversity. The emotion stimulation database built by Lang et al., called the International Affective Picture System (IAPS), contains more than 1000 color images and is currently the most clear content and test–retest reliability database [15]. The use of the IAPS in studies to evoke emotional responses from subjects has received positive evaluations [18, 19]. Therefore, IAPS images were used in this study to induce different emotions in subjects.

In this study, we try to analyze five frequency bands of brain signals by calculating the value of entropy. Entropy is often used to analyze EEG signals [20, 21], and nonlinear statistical analysis methods are used to approximate entropy [22]. The approximate entropy (ApEn) method is often used as the standard method for assessing EEG signals at various frequency bands because it is robust and can quantify EEG signal complexity. With regard to long-term dynamic analysis of EEG signals, the ApEn algorithm can help characterize the dynamics of EEG signals [23]. In addition, permutation entropy (PE) can be also used to eliminate noise, and it has excellent performance in quantifying EEG signal complexity [24, 25]. Azami and Escudero proposed amplitude-aware PE (AAPE), which is more suitable for calculating the EEG signal. It can improve PE and does not consider either the average of the amplitude values or equal amplitude values [26]. Based on the abovementioned discussion of entropy, the ApEn, PE, and AAPE calculation methods were used in this study to evaluate the entropy of different frequencies in EEG signals. Additionally, the analysis of entropy via statistical methods was explored, and the entropy was used as a feature to divide patients into moderately and markedly schizophrenic phases. An identification algorithm was adopted for the training and classification of the feature results. Currently, the well-known identification algorithms include the neural network (NN), support vector machine (SVM), learning vector quantization (LVQ), and other intelligent classifiers. To solve multi-type identification problems, to rapidly establish the available identifier architecture, and to validate the feasibility of the process, the “Classification Learner” application included in MATLAB R2015b (MathWorks, USA) was used for training and classification of the SVM. Currently, this tool is also used in solving EEG signal identification problems [27].

## Methods

### Participants

We classify the participants into three groups: control, moderately, and markedly ill schizophrenic patients. The control (mean age: 41.7 ± 6.31 years; 4 males 6 females), moderately ill, and markedly ill groups were created according to the total scores of PANSS (positive scale, negative scale, and general psychopathology scale). Leucht et al. [28] proposed an accurate definition of the PANSS score (for patients who have a moderately ill patients with baseline PANSS total score of 75, and for the markedly ill patients with a baseline PANSS total score of 95). The scores for moderately ill [mean score of the total PANSS: 70.06 ± 4.25; mean age: 42.18 ± 7.07 years; age of illness onset: 27 ± 8.75 years old; illness duration: 17.53 ± 6.86 years; 8 male 9 female, medication (e.g., chlorpromazine equivalent, mg): 190.59 ± 101.76 mg)] and markedly ill [mean score of the total PANSS: 95.88 ± 10.53; mean age: 42.47 ± 6.98 years; age of illness onset: 23.82 ± 5.74 years old; illness duration: 19.65 ± 6.72 years; 9 male 8 female, medication (e.g., chlorpromazine equivalent, mg): 232.94 ± 91.09 mg)] patients are shown in Table 1.

**Table 1:** Comparison of demographic characteristics between normal, moderately, and markedly ill schizophrenic patients

Demographic variables	Group 1 Normal (*n* = 10)	Group 2 Moderately ill (*n* = 17)	Group 3 Markedly ill (*n* = 17)	Group 1 and 2 (*p* value)	Group 1 and 3 (*p* value)	Group 2 and 3 (*p* value)
Age (years)	41.7 ± 6.31	42.18 ± 7.07	42.47 ± 6.98	.862	.777	.904
Sex (male/female)	4/6	8/9	9/8	0.734	0.534	.741
Age of illness onset (y/o)	0	27 ± 8.75	23.82 ± 5.74	N/A	N/A	.220
Illness duration (years)	0	17.53 ± 6.86	19.65 ± 6.72	N/A	N/A	.370
Medication (e.g., chlorpromazine equivalent, mg)	0	190.59 ± 101.76	232.94 ± 91.09	N/A	N/A	.210
PANSS total	0	70.06 ± 4.25	95.88 ± 10.53	N/A	N/A	.000
PANSS positive	0	17.53 ± 3.28	24.24 ± 4.49	N/A	N/A	.000
PANSS negative	0	16.71 ± 2.57	23.82 ± 4.16	N/A	N/A	.000
PANSS global	0	35.82 ± 4.26	47.82 ± 5.23	N/A	N/A	.000

The participants were recruited from the outpatient clinic at the Department of Psychiatry, Chiayi and Wanqiao Branch, Taichung Veterans General Hospital, Chiayi, Taiwan. The participants underwent screening that included their medical and psychiatric history, laboratory test results, illicit drug screening, and a physical examination. A psychiatric diagnosis of schizophrenia was established using the structured clinical interview from the DSM-IV and a semi-structured interview conducted by a research psychiatrist. After receiving a complete explanation of the study procedures, all participants provided written informed consent as approved by the institutional review board. This study was approved by the ethics committee of the Taichung Veterans General Hospital and was conducted in accordance with Good Clinical Practice procedures and the current revision of the Declaration of Helsinki [29].

### Materials

**Fig. 1:**
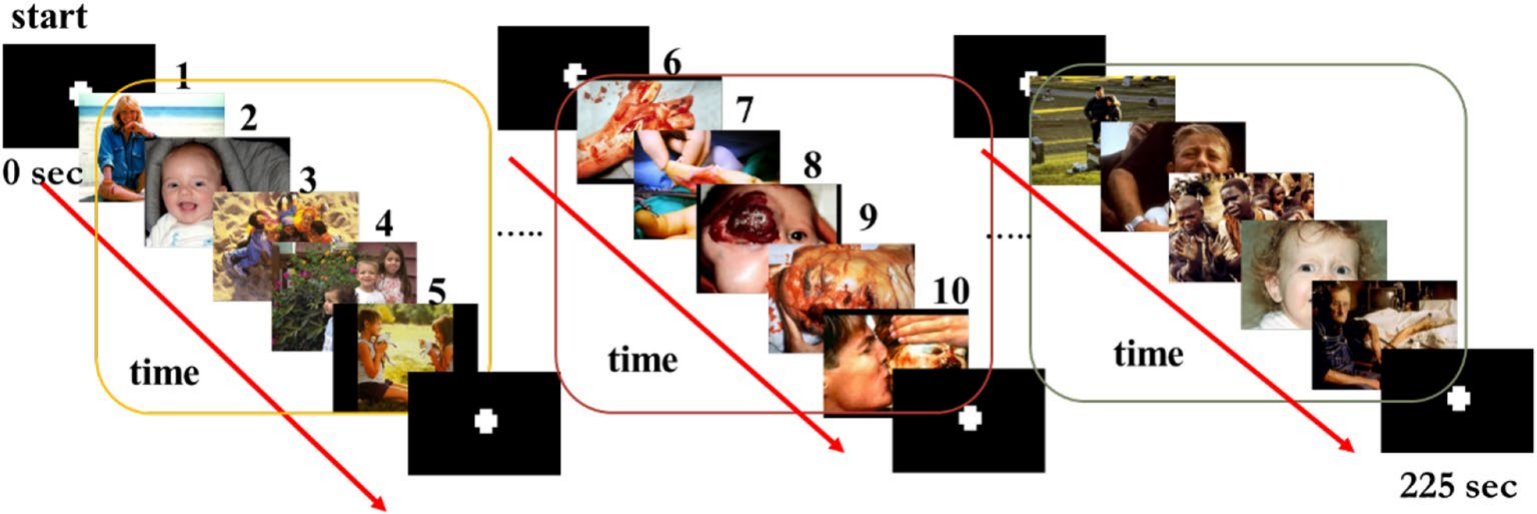
Schematic diagram of the relationship between the time and the order of the images. During each test, each picture was displayed for 3 s, and the patients were allowed 10 s of rest after one stimulation unit that consisted of five pictures of the same emotion. The three types of emotion appeared three times over the course of the entire 225 s test time

In this study, we use three different types of pictures of IAPS as visual stimulations (45 pictures). The study participants [48 males (mean age 35.94 ± 12.38) and 52 females (mean age 37.45 ± 14.14), born and raised in Taiwan] filled out the questionnaires. These emotional pictures were divided into valence and arousal with two dimensions, and the nine-point Likert scale was used for the analysis. The resulting responses to those pictures were divided into three types. Specifically, there are HVLA (high valence low arousal; valence: 7.42 ± 0.51 arousal: 4.77 ± 0.37), LVLA (low valence low arousal; valence: 3.26 ± 0.53 arousal: 4.55 ± 0.86), and LVHA (low valence high arousal; valence: 1.21 ± 0.59 arousal: 6.45 ± 0.56). The relationship between the time and order of the images is shown in Fig. 1. We use three different types of emotional stimulations in this study. Among them, neutral control was not included because the subjects watched emotional pictures prior to the neutral pictures. The continuation time of human moods is different. Thus, we decided not to include the neutral control in this study.

### Entropy calculation method

Entropy is a measure of unpredictability of information content. Therefore, it can display the characterization of the power spectrum. Entropy was first proposed by Shannon in 1940 to measure the unpredictability of information content, and this is now known as information entropy. Subsequently, many similar entropy calculation methods were proposed and applied in the analyses of EEG signals. In 1991, Pincus proposed the ApEn as a technique for measuring sequence complexity and statistical qualification [22]. Most importantly, it measures the probability of generating new models in the signal based on the aspect of measuring the complexity time series. The greater the probability, the more complex the sequence, and the greater the corresponding ApEn. If the EEG signals from a single location are represented as *x*(*i*) = [*u*(*i*), *u*(*i* + 1),…, *u*(*i* + *m* − 1)], *i* = 1, 2,…,(*N* − *m* + 1), where *m* specifies the pattern length and *N* represents the number of time points, then the ApEn definition can be rewritten as follows [22]:

1 $${\text{ApEn}}\left( {m,r,N} \right) = \varPhi^{m} \left( r \right) - \varPhi^{m + 1} \left( r \right),$$

where

2 $$\varPhi^{m} \left( r \right) = \left( {N - m + 1} \right)^{ - 1} \mathop \sum \limits_{i = 1}^{N - m + 1} \log C_{i}^{m} \left( r \right),\;{\text{r represents a predetermined tolerance value}},$$

3 $$C_{i}^{m} \left( r \right) = \frac{{\left( {{\text{number of }}j{\text{ such that d}}\left[ {x\left( i \right),x\left( j \right)} \right] \le r} \right)}}{{\left( {N - m + 1} \right)}},$$

4 $${\text{d}}\left[ {x\left( i \right),x\left( j \right)} \right] = \mathop {\hbox{max} }\limits_{k = 1,2, \ldots ,m} \left( {\left| {u\left( {i + k - 1} \right) - u\left( {j + k - 1} \right)} \right|} \right).$$

PE is a simple type of entropy calculation method that was proposed by Bandt in 2002, and it is able to amplify small detailed changes in the time series [30].

Because of the higher flexibility of the AAPE method compared with the classical PE method in the quantification of signal motifs, it is now straightforward to track changes in both the amplitude and frequency [26]. At the same time, it is important to take into consideration the mean value of amplitudes and the differences between the amplitude values in the AAPE algorithm. When the delay time is set to 1, the definition of PE may be rewritten as follows with *m* as the order of the patterns, *x*_*t*_ the time series, and *N* the time series of length [23]:

5 $${\text{PE}}(m) = - \mathop \sum \limits_{{\pi_{i} = 1}}^{m!} p(\pi_{i} )\,\log p(\pi_{i} ).$$

6 $$p(\pi_{i} ) = \frac{{f(\pi_{i} )}}{N - m + 1},$$

7 $$f(\pi_{i} ) = \left\{ {t|t \le N - m,(x_{t + 1} , \ldots ,x_{t + m} )\;{\text{has type }}\pi_{i} } \right\},$$

where the relative frequency $p(\pi_{i} )$ is the probability of appearance of *π*_*i*_ in various arrangements in the signals. The AAPE method modifies the calculation method for the probability of $p(\pi_{i} )$ by taking the influence of amplitude into consideration to ensure that it is sensitive to the changes in amplitude. When *A* is set to 0.5, the definition of AAPE can be rewritten as follows [26]:

8 $$p_{\text{AAPE}} (\pi_{i} ) = \frac{{ - \mathop \sum \limits_{t = 1}^{N - m + 1} \mathop \sum \limits_{i = 1}^{m!} \left( {\frac{A}{m}\mathop \sum \limits_{k = 1}^{m} \left| {x_{t + (k - 1)} } \right| + \frac{1 - A}{m - 1}\mathop \sum \limits_{k = 2}^{m} \left| {x_{t + (k - 1)} - x_{t + (k - 2)} } \right|} \right)}}{{\mathop \sum \limits_{t = 1}^{N - m + 1} \left( {\frac{A}{m}\mathop \sum \limits_{k = 1}^{m} \left| {x_{t + (k - 1)} } \right| + \frac{1 - A}{m - 1}\mathop \sum \limits_{k = 2}^{m} \left| {x_{t + (k - 1)} - x_{t + (k - 2)} } \right|} \right)}},\;{\text{if type}}\;(X_{t}^{m} ) = \pi_{{_{i} }}^{{}} .$$

9 $${\text{AAPE}}(m) = - \mathop \sum \limits_{{\pi_{i} = 1}}^{m!} p_{\text{AAPE}} (\pi_{i} )\,\log p_{\text{AAPE}} (\pi_{i} ).$$

### EEG signal processing

**Fig. 2:**
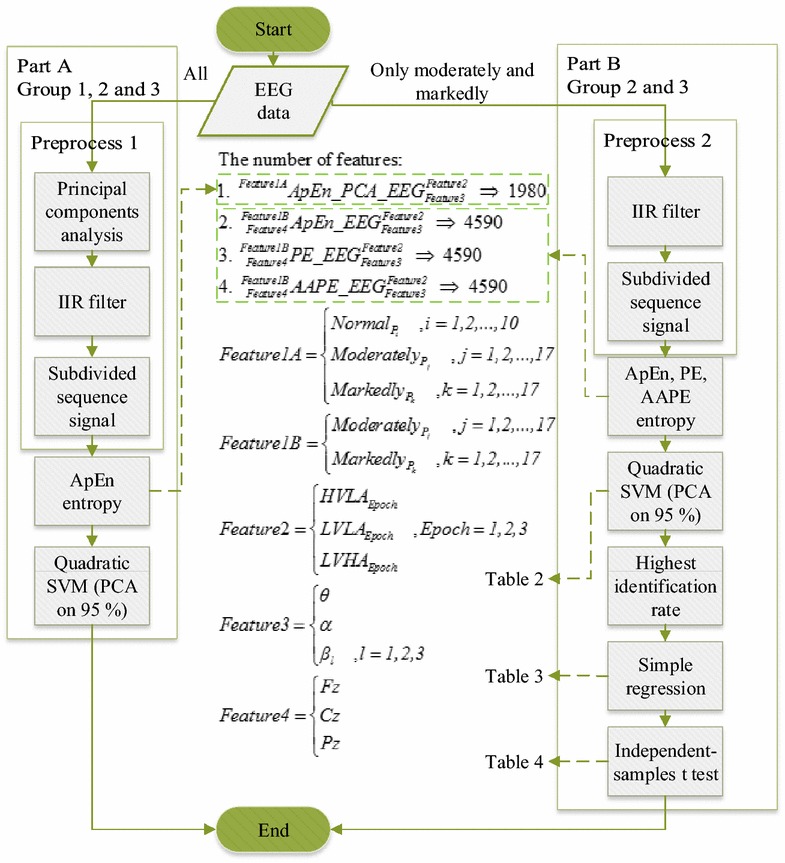
Flowchart of EEG signal processing. First, we wanted to observe the differences between the brainwaves of normal, moderately, and markedly schizophrenic patients. Therefore, we put all signals into a matrix. The PCA method is used to decompose the matrix. Then, the signal is separated into five frequency bands using IIR filters (6-order Butterworth bandpass filters designed with the Matlab R2015b “Signal Processing Toolbox”): *θ*(4–8 Hz), *α*(8–12 Hz), *β*_1_(12–15 Hz), *β*_2_(15–18 Hz), and *β*_3_(18–30 Hz). We separate all different frequency bands of the signals into nine signal fragments according to the timeline that evoked stimuli. Then, we calculate the signals using the ApEn entropy methods. Finally, the obtained features are placed into the SVM (PCA on 95%) for classification. The predictive accuracy was evaluated using the 27-fold cross validation method [32] with a quadratic kernel, in Part A. After that, we classify the features from three points of brainwaves, three types of visual stimuli (HVLA, LVLA, and LVHA), and three methods of entropy (ApEn, PE, and AAPE) in schizophrenic patients. Finally, using linear simple regression and independent-samples *t* test statistical analysis, we analyze the features of the highest identification degree in the classification results and the total scores of PANSS, in Part B

The brainwaves were captured using a Procomp Infiniti™ (Thought Technology Ltd, Montreal West, Quebec, Canada). The sampling rate was 256 Hz, the Fz, Cz, and Pz locations were used as electrode points. The power source has four 1.5 V AA batteries, thus the frequency noise of alternating current does not exist. Left ear (A1), right ear (A2), and (A1 + A2)/2 were used for the reference electrode. According to the studies of Huang et al. [31], the electrode sites were identified by Fp1, Fp2, AF3, AF4, F7, F3, Fz, F4, F8, FC5, FC1, FC2, FC6, T7, C3, Cz, C4, T8, CP5, CP1, CP2, CP6, P7, P3, Pz, P4, P8, PO3, PO4, O1, and O2. However, we found that the used statistical method with factor analysis supports electrode positions. Specifically, Fz, Cz, and Pz were indicators of schizophrenia. Therefore, Fz, Cz, and Pz electrode positions are used in this study. To avoid the sound and light interferences that happen during the experimental procedure, the room was surrounded by three layers of curtains. The subjects only needed to focus on the front visual-stimulation video during the test. They needed to prevent any unnecessary movements to avoid unnecessary noise. We stopped the test if the subjects felt uncomfortable. IAPS images in a specific time sequence were shown to the subjects while the EEG signals from Fz, Cz, and Pz points were recorded. To explore the reaction of brainwave, we proposed an EEG signal processing procedure, as shown in Fig. 2.

## Results

### Classification of different groups based on brainwaves

We used the ApEn method to calculate the preprocessing signals as features for the classification. For the normal and markedly ill groups, the identification rate was 81.5%. For the normal and moderately ill groups, the identification rate was 70.4%. For the markedly and moderately ill groups, the identification rate was 67%.

According to the abovementioned results, we observed the differences between the markedly and moderately ill groups. Table 2 shows the classification of moderately and markedly ill schizophrenic patients using the features calculation methods at the Fz, Cz, and Pz points. The results indicate that the identification rate at the Fz point was the highest when using the ApEn method, and the identification rate was as high as 79.5% for the moderately and markedly ill schizophrenic patients under the HVLA stimulation.

**Table 2:** Identification rates of entropy and emotion at different points (%)

Brainwave point	Entropy	Type			Avg. (%)
		HVLA (%)	LVLA (%)	LVHA (%)	
Fz	ApEn	79.5	73.5	73.5	75.5
	PE	52.9	73.5	58.8	61.7
	AAPE	64.7	64.7	70.6	66.7
Cz	ApEn	41.2	35.3	58.8	45.1
	PE	38.2	50	50	46.1
	AAPE	41.2	32.4	52.9	42.2
Pz	ApEn	38.2	50	58.8	49
	PE	67.6	55.9	73.5	65.7
	AAPE	52.9	67.6	61.8	60.8

### Correlation analysis between the features of each frequency bands and the total scores of PANSS

The total scores in the PANSS test can specifically evaluate thoughts. As described above, for the signals recorded at the Fz point, the ApEn method is the best method to use to identify moderate and markedly ill schizophrenic patients. Therefore, the correlations test was performed based on the total scores of PANSS and the frequency bands that were calculated using the signals captured at the Fz point and processed using the ApEn method. The results are summarized in Table 3. The results show that the *p* value of the correlation analysis at *β*_1_(12–15 Hz) and *β*_2_(15–18 Hz) was less than .05, which indicates that the features of these frequencies significantly affect the identification.

**Table 3:** Correlation analysis between the total scores of PANSS and the frequency bands calculated using the ApEn method at the Fz point

	Type								
	HVLA			LVLA			LVHA		
Section:	1	2	3	1	2	3	1	2	3
Frequency									
*θ*(4–8 Hz)									
Pearson’s *r*	.071	.153	.202	.023	.149	.43	−.070	.038	.088
*p* value	.692	.387	.252	.897	.401	.809	.696	.831	.620
*α*(8–12 Hz)									
Pearson’s *r*	.224	.170	.188	.169	.182	.160	.130	.082	.094
*p* value	.202	.337	.288	.339	.304	.367	.464	.645	.595
*β*_1_(12–15 Hz)									
Pearson’s *r*	*.366*	*.472*	*.472*	*.366*	*.542*	*.505*	*.346*	*.454*	*.382*
*p* value	.033*	.005**	.005**	.033*	.001**	.002*	.045*	.007*	.026**
*β*_2_(15–18 Hz)									
Pearson’s *r*	*.553*	*.548*	*.590*	*.582*	*.569*	*.555*	*.554*	*.561*	*.573*
*p* value	.001**	.001**	.000***	.000***	.000***	.001**	.001**	.001**	.000***
*β*_3_(18–30 Hz)									
Pearson’s *r*	−.248	−.263	−.257	−.302	−.234	−*.342*	−2.37	−.339	−.230
*p* value	.157	.133	.142	0.83	.182	.048*	.176	.050	.191

* *p* < .05, ** *p* < .01, *** *p* < .001

### Independent-samples *t* test between the features of beta frequency bands and the group 2 and 3

Based on the above results, there were significant differences in beta frequency bands. Therefore, using the features of beta frequency bands, the group 2 and 3 (moderately and markedly ill schizophrenic patients) were analyzed with independent-samples *t* test. The results are summarized in Table 4. The results show a significant difference between the group 2 and 3 at *β*_1_(12–15 Hz) and *β*_2_(15–18 Hz).

**Table 4:** Independent-samples *t* test between the group 2 and 3 and beta frequency bands calculated using the ApEn method at the Fz point

	Type								
	HVLA			LVLA			LVHA		
Section:	1	2	3	1	2	3	1	2	3
Frequency									
*β*_1_(12–15 Hz)									
Moderately ill	.583	.597	.589	.587	.587	.590	.588	.587	.573
Mean (SD)	(.069)	(.040)	(.048)	(.055)	(.042)	(.043)	(.050)	(.046)	(.062)
Markedly ill	.625	.624	.627	.626	.627	.627	.621	.624	.622
Mean (SD)	(.009)	(.006)	(.005)	(.005)	(.004)	(.005)	(.025)	(.016)	(.018)
*t* value	−2.462	−2.801	−3.231	−2.860	−3.999	−3.616	−2.417	−3.126	−3.086
*p* value	.025*	.012*	.005**	.011*	.001**	.002**	.024*	.005**	.006**
*β*_2_(15–18 Hz)									
Moderately ill	.538	.543	.540	.538	.540	.540	.537	.536	.537
Mean (SD)	(.071)	(.067)	(.067)	(.064)	(.067)	(.065)	(.064)	(.067)	(.066)
Markedly ill	.608	.606	.609	.606	.606	.605	.602	.605	.607
Mean (SD)	(.013)	(.012)	(.008)	(.006)	(.013)	(.011)	(.016)	(.021)	(.014)
*t* value	−4.050	−3.812	−4.214	−4.325	−3.957	−4.014	−4.047	−4.052	−4.276
*p* value	.001**	.001**	.001**	.001**	.001**	.001**	.001**	.001**	.000***
*β*_3_(18–30 Hz)									
Moderately ill	.564	.575	.568	.569	.567	.570	.568	.573	.563
Mean (SD)	(.051)	(.044)	(.043)	(.047)	(.045)	(.045)	(.046)	(.045)	(.051)
Markedly ill	.554	.555	.557	.554	.558	.555	.556	.554	.552
Mean (SD)	(.015)	(.015)	(.015)	(.014)	(.012)	(.013)	(.019)	(.018)	(.014)
*t* value	.788	1.735	1.041	1.275	.821	1.381	.960	1.581	.897
*p* value	.441	.092	.310	.218	.422	.184	.344	.124	.381

* *p* < .05, ** *p* < .01 *** *p* < .001

## Discussion

EEG signals are complex nonlinear dynamic signals, and it is challenging to accurately extract the EEG signal characteristics. In this study, images from the IAPS were used to evoke emotional responses from subjects while the corresponding EEG signals were recorded. Then, the EEG signals at different frequency bands were analyzed, and different entropy calculation methods were used to explore the differences between normal, moderately, and markedly ill schizophrenic patients.

Based on the identification results, we can clearly determine that normal and schizophrenic patients have significant differences in the reaction of brainwaves. Therefore, further discussion about the schizophrenic patients in different situations is pending. PANSS is a medical scale for measuring symptom severity of schizophrenic patients. We separated schizophrenic patients into moderately and markedly ill based on the total scores of PANSS, and then calculated the features from the proposed preprocessing approach. The identification results are shown in Table 2. Firstly, we observed that the highest average identification rate was 75.5%, which was calculated using the ApEn method of the Fz point signal. Fz points belong to the frontal area. Therefore, this result corresponds with the research of fMRI [33, 34] and EEG [35, 36]. Then, for different types of emotion, the identification rate was 79.5% in HVLA, and 73.5% in LVHA and LVHA. The results showed that the calculated ApEn features at the Fz point when combined with the SVM identification can effectively classify moderately and markedly ill patients. This confirms that the differences in the EEG signal responses at the Fz point among the patients with different emotional stimulations were significant, and ApEn as features can be simplified for clear identification.

According to the abovementioned results, the ApEn features calculated from the Fz point is the best method for identifying moderately and markedly ill schizophrenic patients, and the total scores of the PANSS test can be specifically evaluated. Further, the correlation between the Fz point signal at five frequency bands and the total scores of PANSS was evaluated. The signals, which are separated into five frequency bands, are considered to be functionally significant by many researchers and correspond to general characteristics. Specifically, Theta is creativity, insight, deep states; Alpha is alertness, peacefulness, readiness; Beta is thinking, focusing, sustained attention [37]. In addition, some researchers divide Beta into three bands for observation and analysis [38]. It can be considered abnormal and leads to several neural diseases [39]. Table 3 shows that the *p* value for the features extracted from the *β*_1_(12–15 Hz) and *β*_2_(15–18 Hz) signals at the Fz point and the total scores of PANSS were less than .05 and .001, respectively, which indicates that there was some correlation between the features extracted from the signals at this frequency and the scale score. The result from Table 4 also showed that there were significant differences between the moderately and markedly ill schizophrenic patients at *β*_1_(12–15 Hz) and *β*_2_(15–18 Hz). The Beta wave tends to exhibit larger changes under external stimulation, and hence, with visual emotional stimulation, the features obtained from the Beta are able to better represent the differences between patients with different degrees of illness by applying visual emotional stimulation on schizophrenic patients.

## Conclusions

The IAPS were used to evoke three different types of emotions in schizophrenic patients while the corresponding EEG signals were recorded. The SVM algorithm was used as the classifier for normal, moderately, and markedly ill schizophrenic patients. The identification result was as high as 81.5%. We further explore and classify moderately and markedly ill schizophrenic patients. The identification rate of the ApEn features calculated for the HVLA at the Fz point was as high as 79.5%. Thus, ApEn is better than PE and AAPE entropy calculation method. The correlation analysis between the ApEn features at the Fz point and the total scores of the PANSS test show that the *p* value was less than .001 at the beta band (15–18 Hz frequency range). The results indicate that the signal analysis method proposed in this study can provide reference information that can be used to determine the phases of schizophrenia symptoms in clinical applications.

## Abbreviations

EEG: electroencephalography
IAPS: International Affective Picture System
IIR filter: infinite impulse response filter
PANSS: positive and negative syndrome scale
SVM: support vector machine
HVLA: high valence low arousal
LVLA: low valence low arousal
LVHA: low valence high arousal
